# Synaptotagmin-2 Is a Reliable Marker for Parvalbumin Positive Inhibitory Boutons in the Mouse Visual Cortex

**DOI:** 10.1371/journal.pone.0035323

**Published:** 2012-04-23

**Authors:** Jean-Pierre Sommeijer, Christiaan N. Levelt

**Affiliations:** Department of Molecular Visual Neuroscience, Netherlands Institute for Neuroscience, an institute of the Royal Netherlands Academy of Arts and Sciences, Amsterdam, The Netherlands; Virginia Commonwealth University Medical Center, United States of America

## Abstract

**Background:**

Inhibitory innervation by parvalbumin (PV) expressing interneurons has been implicated in the onset of the sensitive period of visual plasticity. Immunohistochemical analysis of the development and plasticity of these inhibitory inputs is difficult because PV expression is low in young animals and strongly influenced by neuronal activity. Moreover, the synaptic boutons that PV neurons form onto each other cannot be distinguished from the innervated cell bodies by immunostaining for this protein because it is present throughout the cells. These problems call for the availability of a synaptic, activity-independent marker for PV+ inhibitory boutons that is expressed before sensitive period onset. We investigated whether synaptotagmin-2 (Syt2) fulfills these properties in the visual cortex. Syt2 is a synaptic vesicle protein involved in fast Ca^2+^ dependent neurotransmitter release. Its mRNA expression follows a pattern similar to that of PV throughout the brain and is present in 30–40% of hippocampal PV expressing basket cells. Up to now, no quantitative analyses of Syt2 expression in the visual cortex have been carried out.

**Methodology/Principal Findings:**

We used immunohistochemistry to analyze colocalization of Syt2 with multiple interneuron markers including vesicular GABA transporter VGAT, calbindin, calretinin, somatostatin and PV in the primary visual cortex of mice during development and after dark-rearing.

**Conclusions/Significance:**

We show that in the adult visual cortex Syt2 is only found in inhibitory, VGAT positive boutons. Practically all Syt2 positive boutons also contain PV and vice versa. During development, Syt2 expression can be detected in synaptic boutons prior to PV and in contrast to PV expression, Syt2 is not down-regulated by dark-rearing. These properties of Syt2 make it an excellent marker for analyzing the development and plasticity of perisomatic inhibitory innervations onto both excitatory and inhibitory neurons in the visual cortex.

## Introduction

Ocular dominance plasticity is a classical model for studying experience-dependent plasticity in the neocortex. When during a sensitive period of development vision in one eye is deprived (monocular deprivation, MD) the visual cortex becomes less responsive to this eye, while responsiveness to the open eye strengthens. It is now well established that the onset of the sensitive period of OD plasticity is regulated by the development of inhibitory innervation [1], [2]. Especially the maturation of synapses formed by parvalbumin (PV) positive interneurons seems to determine the start of the sensitive period [3]. This maturation is dependent on visual experience, and is regulated by BDNF and transsynaptic transportation of the transcription factor OTX-2 from the retina to PV+ interneurons in the visual cortex [3].

How PV+ interneurons affect OD plasticity is under intense investigation. Most of these cells are fast-spiking large basket cells. They predominantly innervate the somata and proximal dendrites of excitatory [4] and inhibitory neurons [5] where they are at the perfect position to alter spike timing dependent plasticity by blocking dendritic back-propagation. Moreover, PV+ interneurons form networks connected through reciprocal chemical and electrical synapses [6], [7] which allows them to coordinate the firing of large sets of cortical excitatory neurons. This underlies the appearance of gamma-band oscillations (30–100 Hz) [8] believed to be involved in the processing of sensory input [9], attention and experience-dependent plasticity [10], [11].

In order to study the experience-dependent development and plasticity of synaptic boutons formed by PV+ interneurons, tools to visualize these structures are essential. Although antibodies to PV can be used for this purpose, this approach has three important drawbacks: i) In the visual cortex, PV expression in synaptic boutons only becomes detectable after approximately three weeks of age, [12], ii) PV expression is activity dependent [13]–[16], and iii) PV is expressed throughout the cell. These properties make it difficult to distinguish activity- or age-dependent formation of PV+ synapses from changes in PV expression, and complicate the visualization of PV+ boutons forming synapses onto other PV+ interneurons. To circumvent these problems, we searched for a synaptic, activity-independent marker that is expressed before sensitive period onset.

One such potential marker is synaptotagmin-2 (Syt2). Syt2 is a synaptic vesicle membrane protein involved in fast, Ca^2+^ dependent neurotransmitter release [17]–[23]. It has been suggested to be specific to inhibitory neurons based on its scattered localization and its presence in the striatum and the reticular nucleus where most neurons are inhibitory [22]. Moreover, Syt2 is highly expressed in perisomatic synapses [24] and is localized in many PV-expressing presynaptic boutons in hippocampal cultures [25]. However, quantitative studies assessing whether Syt2 expression is restricted to PV+ interneurons in the cortex or whether it can also be found in other subsets of excitatory or inhibitory neurons are missing, as are studies assessing the effects of visual deprivation on Syt2 expression.

We therefore investigated the colocalization of Syt2 with various markers for interneuron-subsets and their synapses. We find that Syt2 and PV show almost complete co-expression in inhibitory boutons of the mouse visual cortex, that Syt2 expression is detectable well before sensitive period onset and that dark rearing does not down-regulate its expression.

## Results

### Syt2 and PV have similar expression patterns in the adult mouse brain

**Figure 1:**
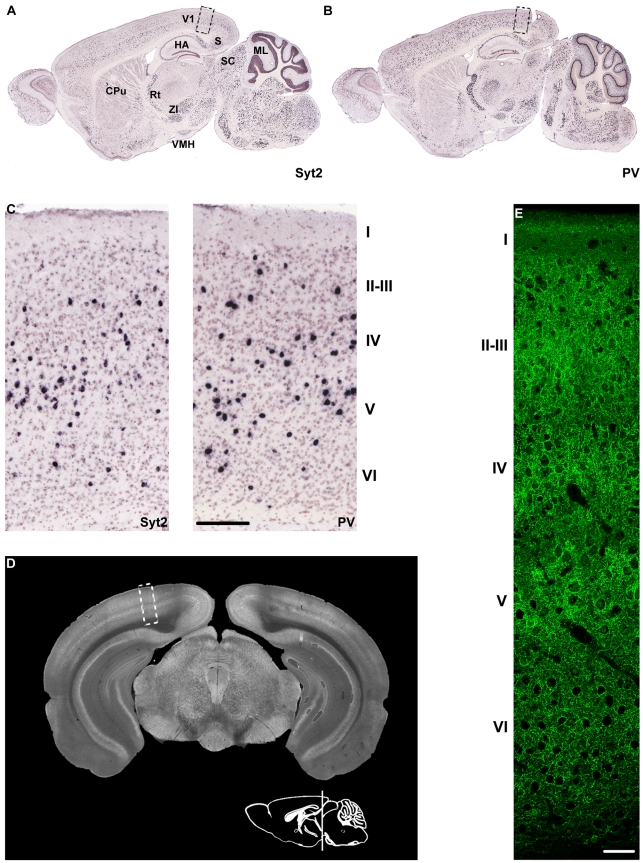
Syt2 and PV expression in the mouse brain. (A–C) Localization of Syt2 and PV mRNA in the mouse brain according to the Allen Brain Atlas [26]–[27]. Note the resemblance between the two patterns. Syt2 mRNA is detectable in the Caudate Putamen (CPu), reticular thalamic nucleus (Rt), zona incerta (ZI) and ventromedial hypothalamic nucleus (VMH). Expression is also seen in the hippocampal areas and the subiculum, as well as in the superior colliculus (SC) and the molecular layer of the cerebellum (ML). A sparse but layered signal is seen in the visual cortex (V1) (C). (D) Syt2 protein expression is visualized in a coronal section of the mouse brain using the i735 antibody. The white box highlights the part of the visual cortex from which data for figures 2–4 and 7&8 are obtained. (E) Syt2 is expressed in all layers of the visual cortex, except for layer 1. It is localized in a perisomatic fashion around many cells in L2-3 – L6. Expression is strongest in the lower part of L5 where larger pyramidal neurons reside. Scale bar (C) 180 µm, (E) 50 µm.

We first investigated the expression pattern of Syt2 mRNA in the mouse forebrain making use of the Allen Brain Atlas [26], [27], and compared it to the expression of PV mRNA. We found that the expression patterns were strikingly similar (Fig. 1A, B, C). mRNA expression patterns of both genes in the neocortex, the hippocampal areas (HA) and subiculum (S) were sparse and scattered, typical for interneuron-specific expression. Syt2 expression was dense in striatal caudate putamen, the reticular thalamic nucleus, the zona incerta and the ventromedial hypothalamic nucleus, as well as in the superior colliculus and the molecular layer of the cerebellum [22]. This expression pattern is very similar to that of PV.

We next analyzed the expression of Syt2 protein using an antibody generated against residues 81–422 of Syt2 [17] (a kind gift from Dr. T. Südhof) (Fig. 1D). Since Syt2 is a presynaptic protein and not present in cell bodies, expression was more diffuse and widespread than its mRNA. In coronal sections, Syt2 was found to be expressed in layers 2–6 of the visual cortex. At higher magnification (Fig. 1E) Syt2 was found to be localized perisomatically. Especially large neurons in L5 were extensively innervated by Syt2 positive puncta. These results show that Syt2 is expressed at the mRNA and protein level as would be expected for a presynaptic protein present in PV expressing interneurons.

### Syt2 in mouse visual cortex is specifically expressed in PV positive boutons

**Figure 2:**
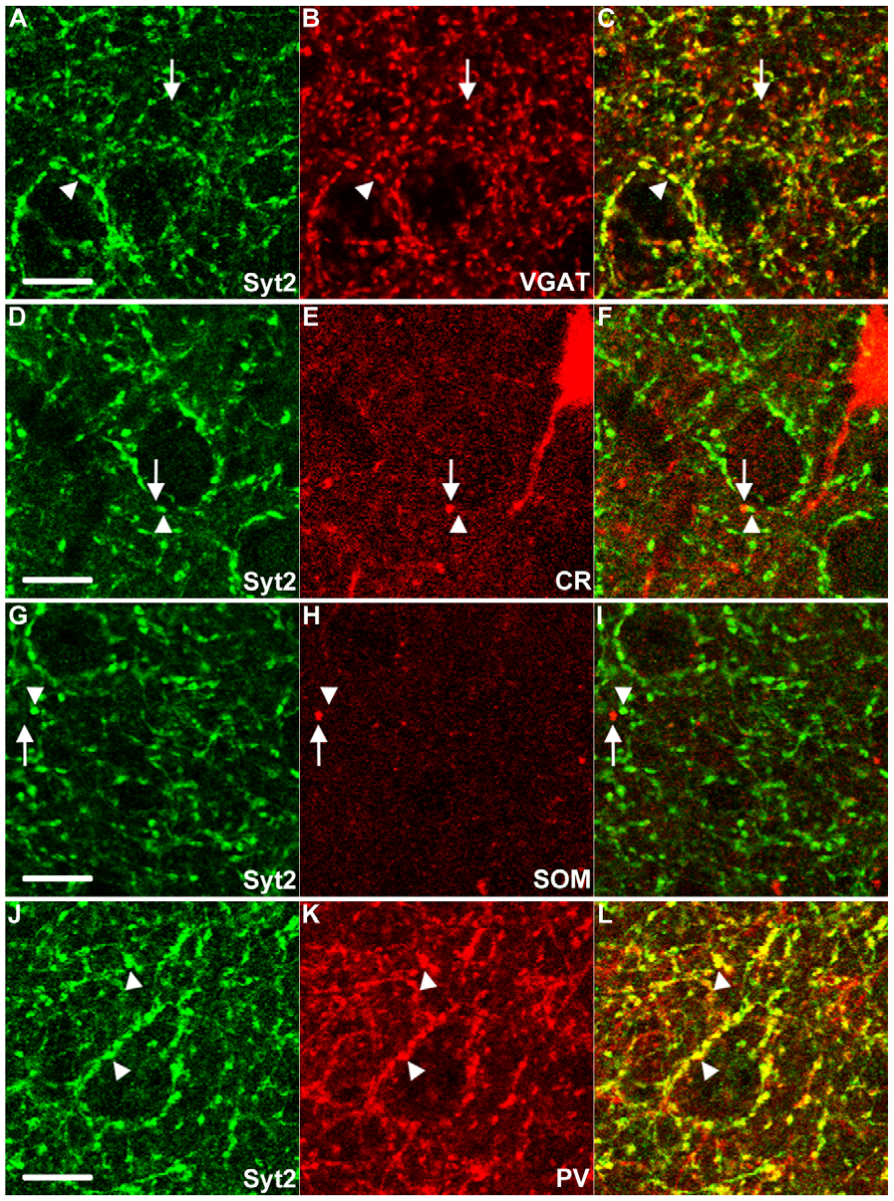
Syt2 in L2-3 is expressed in PV positive boutons. (A–C, arrowhead) Syt2 almost always colocalizes with VGAT in L2-3 of the visual cortex. The arrow indicates a VGAT positive bouton without Syt2 expression. (D–I) Syt2 puncta (arrowhead) are not calretinin (CR, arrow) or somatostatin (SOM, arrow) positive. (J–L) Syt2 shows strong co-expression with PV (arrowheads). A–C & G–I n = 3; D–F & J–L n = 4. Scale bars 10 µm.

**Figure 3:**
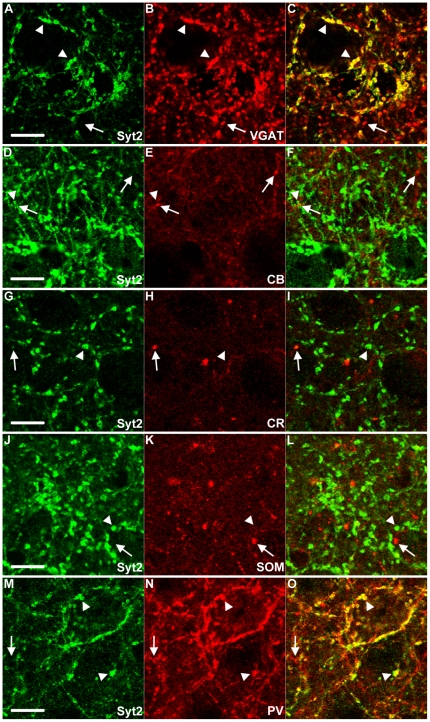
Syt2 in L5 is expressed in PV positive boutons. (A–C, arrowheads) Syt2 almost always colocalizes with inhibitory VGAT positive puncta in L5 of the visual cortex. The arrow indicates a VGAT positive punctum without Syt2 expression. (D–L) These inhibitory Syt2 puncta (arrowheads) are not calbindin positive (D–F), CR positive (G–I) or SOM positive (J–L) (arrows). (M–O) As in L2-3, Syt2 shows a high colocalization with PV positive puncta in L5 of the visual cortex (arrowheads). Note the PV+ neurite (arrow) which is without expression of Syt2 (M–O). A–C & J–L n = 3; D–I & M–O n = 4. Scale bars 10 µm.

**Figure 4:**
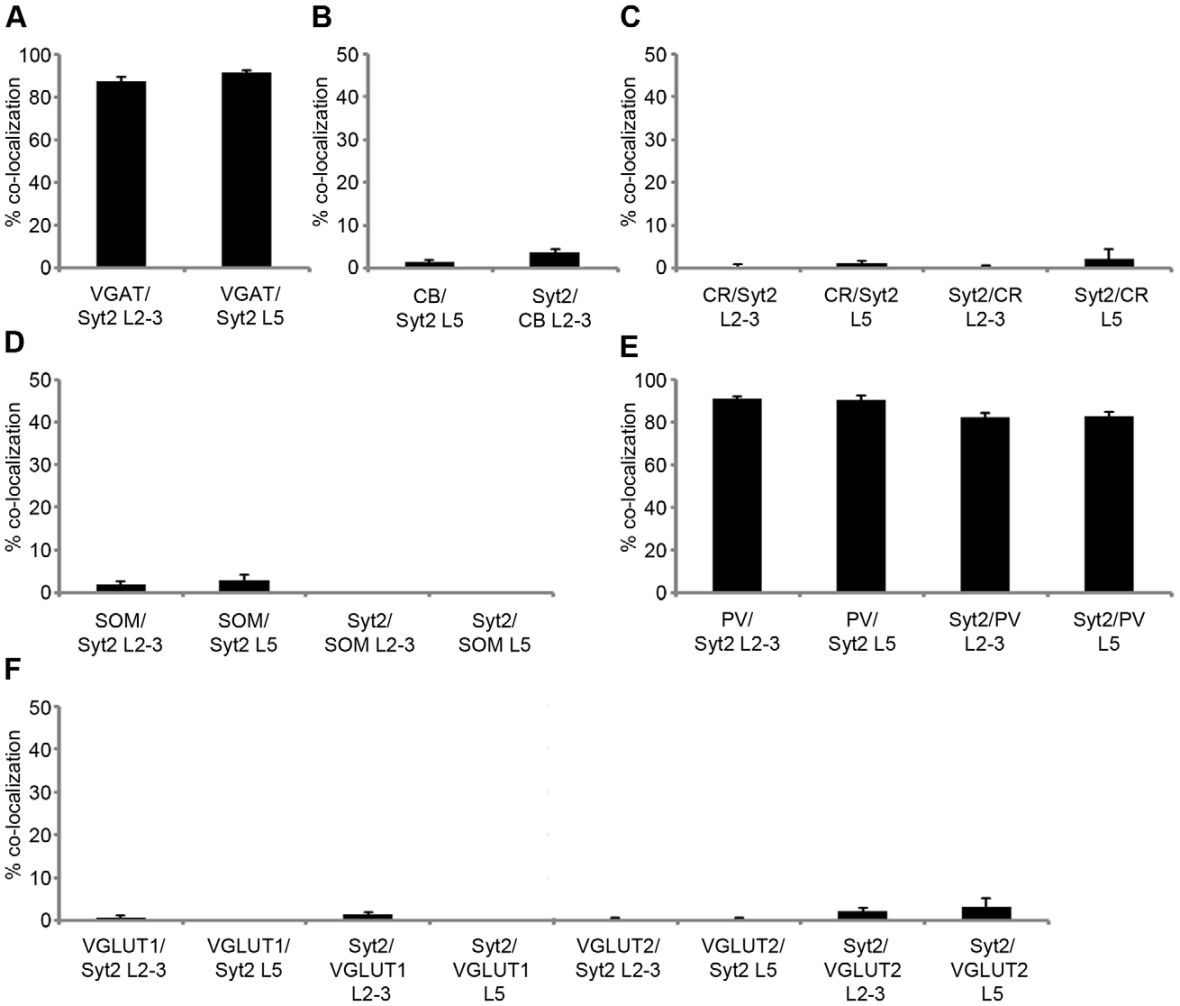
Syt2 in L2-3 and L5 is not expressed in VGLUT1&2 positive boutons. (A–F) Syt2 puncta (arrowheads) in L2-3 and L5 are not VGLUT1 positive. Arrows indicate VGLUT1 boutons. VGLUT1 and Syt2 show an alternating perisomatic localization (F). Similarly VGLUT2 boutons, indicated with arrows do not show expression of Syt2. Syt2 puncta indicated with arrowheads (G–L). A–L n = 3. Scale bars 10 µm.

To confirm that Syt2 was specifically expressed in boutons of inhibitory neurons in the visual cortex, we examined its colocalization with the vesicular GABA transporter (VGAT), a marker for most types of inhibitory synapses [28]–[30]. We found that 87% and 91% of all Syt2 puncta showed co-expression of VGAT in L2-3 (Fig. 2A, B, C) and L5 (Fig. 3A, B, C) respectively, confirming expression of Syt2 in inhibitory synapses (Fig. 4A). Because we noticed that the Syt2+ puncta that were scored as negative for VGAT showed VGAT expression in the upper range of the background levels (not shown), it is well possible that the reported percentages are an underestimation of the actual level of colocalization.

**Figure 5:**
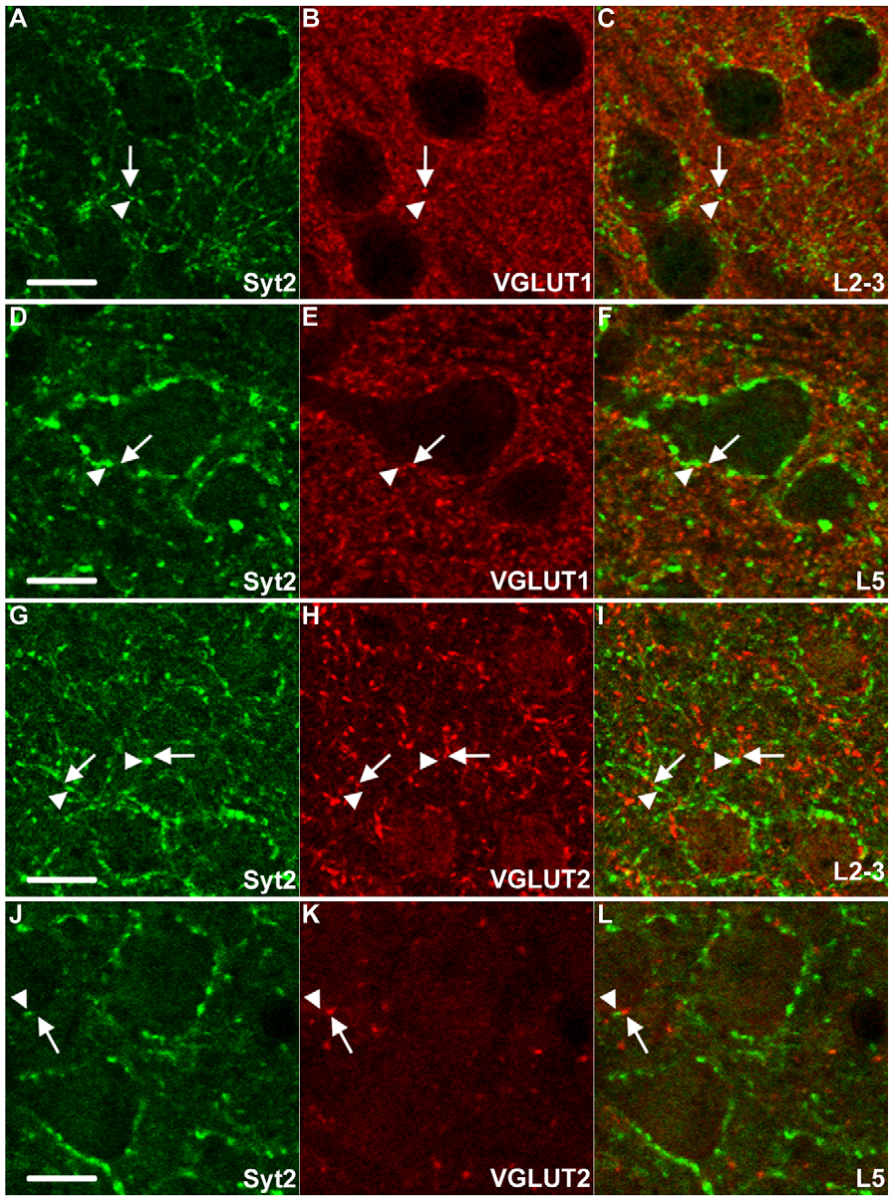
Quantification of colocalization of Syt2 and markers for specific subsets of inhibitory and excitatory neurons. (A) Percentage of colocalization of VGAT in Syt2+ puncta in L2-3 and L5. (B) Percentage of calbindin colocalization in Syt2+ puncta (CB/Syt2), and Syt2 in calbindin+ puncta (Syt2/CB) in L5. (C) Percentage of colocalization of calretinin in Syt2+ puncta (CR/Syt2) and Syt2 in calretinin+ puncta (Syt2/CR). (D) Percentages colocalization of somatostatin in Syt2+ puncta (SOM/Syt2) and Syt2 in somatostatin+ puncta (Syt2/SOM). (E) Percentages colocalization of PV in Syt2+ puncta (PV/Syt2) and Syt2 in PV+ puncta (Syt2/PV). (F) Percentages colocalization of VGLUT1 or VGLUT2 in Syt2+ puncta (VGLUT1/Syt2 and VGLUT2/Syt2) and Syt2 in VGLUT1+ or VGLUT2+ puncta (Syt2/VGLUT1 and Syt2/VGLUT2). Data are given in mean percentage ± SEM.

Next we wanted to know whether Syt2 was expressed in a specific subset of interneurons. We used antibodies against four other markers for inhibitory interneurons, calbindin, calretinin, somatostatin and PV. In Table 1, the exact percentages of puncta with colocalization of Syt2 and these interneuron markers are shown. We found that the expression of calbindin in the upper layers of the cortex was extensive but diffuse, probably due to weak expression of calbindin in excitatory neurons. This made the assessment of co-expression with Syt2 impossible in these layers. In L5 of the visual cortex, however, calbindin expression was restricted to inhibitory neurons [31], [32] and clear puncta could be identified (Fig. 3D, E, F). In this layer less than 2% of all Syt2 puncta were calbindin positive and less than 4% of calbindin puncta were Syt2 positive (Fig. 3D, E, F, Fig. 4B). There was also negligible colocalization of calretinin with Syt2 in L2-3 and L5. In both L2-3 and L5 almost no (0.5% and 1% respectively) Syt2 puncta were positive for calretinin and Syt2 was hardly (0.4% and 2.3% respectively) found in calretinin positive puncta (Fig. 2D, E, F, Fig. 3G, H, I, Fig. 4C). Antibodies to somatostatin labeled 1.9% of Syt2 puncta in L2-3 and 2.9% in L5, and a negligible amount of somatostatin positive puncta in L2-3 and L5 were positive for Syt2 (Fig. 2G, H, I, Fig. 3J, K, L, Fig. 4D). Thus, only a very small fraction of Syt2 puncta is also positive for calbindin, calretinin or somatostatin. In strong contrast, antibodies to PV labeled over 90% of Syt2 puncta in L2-3 and L5 and 82% of all PV puncta were also positive for Syt2 in L2-3 and L5 (Fig. 2J, K, L, Fig. 3M, N, O, Fig. 4E. Also Fig. S2, S3). Similar colocalization percentages were observed for L4 and L6 (Fig. S1, Table 1). PV is a soluble Ca^2+^-binding protein that is present throughout the neuron. PV is therefore also present in the entire dendrites and axon. This may partially explain why not all PV puncta were Syt2 positive (Fig. 4E), as these include tangentially cut dendrites and axons that do not represent presynaptic boutons. When immunolabeled for both PV and Syt2 it was clear that Syt2 and PV colocalized in puncta only and that Syt2 was not present in any stretches of neurites that were PV positive (Fig. S2A, B, C). Within the white-dashed boxes, figure S2B shows both PV positive puncta as well as a stretch of neurite labeled for PV. This neurite was devoid of Syt2 labeling, while there was clear colocalization within PV positive puncta (Fig. S2C). Restricted localization of Syt2 in presynaptic boutons is also supported by the fact that Syt2 was present specifically in VGAT positive boutons (Fig. 2A, B, C, Fig. 3 A, B, C). To rule out any expression of Syt2 in glutamatergic puncta we investigated the colocalization of the vesicular glutamate transporters 1 and 2 (VGLUT1&2). As expected, in both L2-3 and L5 practically no VGLUT1 was expressed in Syt2 puncta (0.6% and 0% respectively) and very few VGLUT1 puncta were positive for Syt2 (1.3% and 0% respectively, Fig. 5A, B, C, D,E, F, Fig. 4F). A similar distribution could be seen for Syt2+ puncta expressing VGLUT2 (0.5% and 0.3% in L2-3 and L5, Fig. 5G, H, I, J, K, L, Fig. 4F), and VGLUT2+ puncta expressing Syt2 (2.1% in L2-3 and 3.2% in layer 5). We conclude that almost all PV positive boutons in the visual cortex express Syt2 and vice versa.

**Table 1:** Percentages of colocalization of Syt2 with different markers.

	% co-localization:	n:	% co-localization:	n:
Immunolabeled:	L2-3		L5	
Syt2 puncta positive for VGAT	87.3%±2.1%	767	91.2%±1.4%	966
Syt2 puncta positive for calbindin	n/a	n/a	1.4%±0.5%	1213
Calbindin puncta positive for Syt2	n/a	n/a	3.7%±0.8%	806
Syt2 puncta positive for calretinin	0.5%±0.4%	408	1.0%±0.6%	323
Calretinin puncta positive for Syt2	0.4%±0.4%	356	2.3%±2.3%	224
Syt2 puncta positive for somatostatin	1.9%±0.9%	818	2.9%±1.3%	578
Somatostatin puncta positive for Syt2	0.1%±0.1%	616	0	547
Syt2 puncta positive for PV	90.7%±1.3%	766	90.4%±1.7%	680
PV puncta positive for Syt2	82%±2.1%	798	82%±1.8%	768
Syt2 puncta positive for VGLUT1	0.6%±0.6%	380	0	235
VGLUT1 puncta positive for Syt2	1.3%±0.7%	354	0	236
Syt2 puncta positive for VGLUT2	0.5%±0.3%	385	0.3%±0.3%	290
VGLUT2 puncta positive for Syt2	2.1%±0.8%	420	3.2%±2.0%	350
	L4		L6	
Syt2 puncta positive for PV	91.0%±1.6%	360	90.9%±2.1%	358
PV puncta positive for Syt2	87.5%±1.2%	367	81.3%±2.3%	322

Values represent e.g. the mean percentages (± SEM) of Syt2 puncta that also expressed VGAT.

### Syt2 is specifically expressed in PV positive boutons in other cortical areas

**Figure 6:**
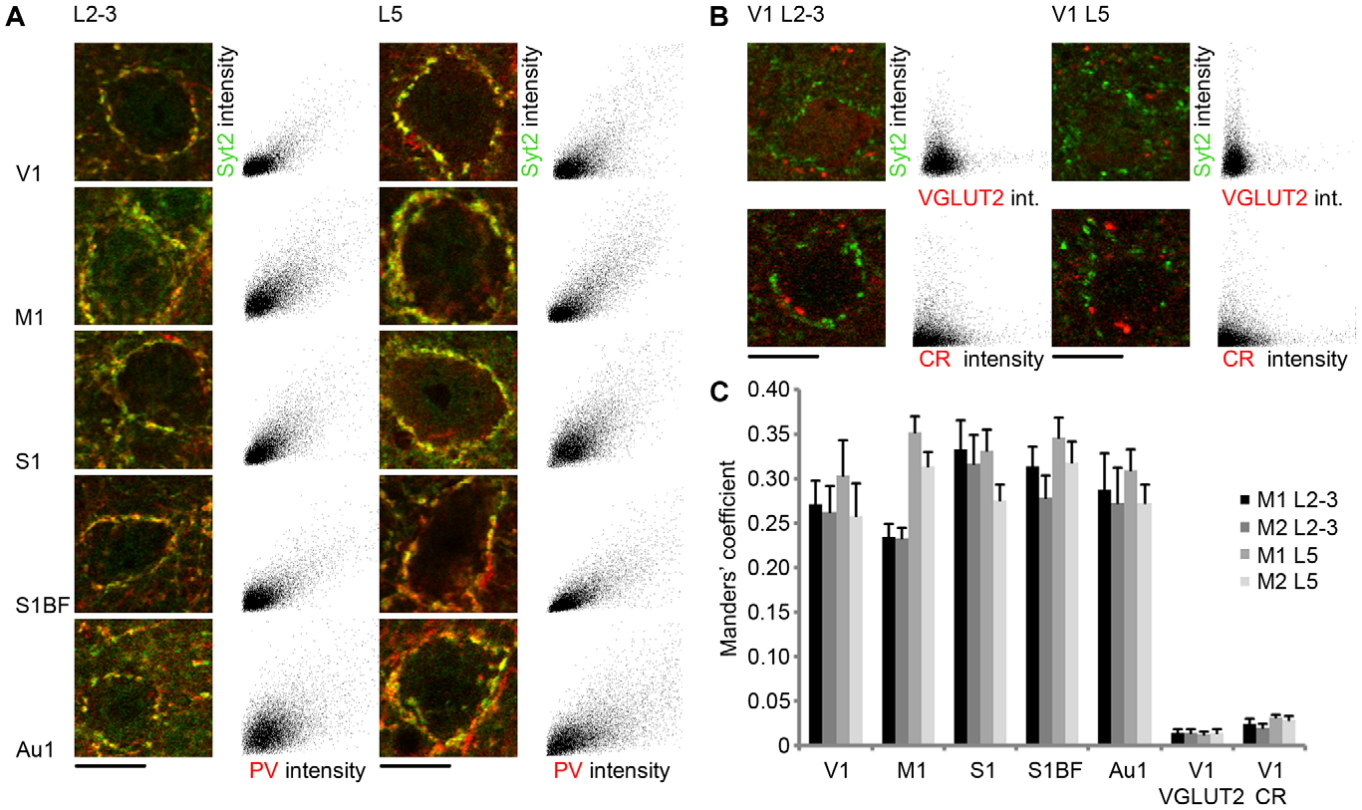
Scatter plot pixel analyses of Syt2 colocalization with cell type specific markers in various cortical areas. (A) Example images of neurons with perisomatic punctate innervation from different areas of the cortex. Adjacent are scatter plots for the same images showing distribution of fluorescence intensities for Syt2 (vertical green channel) and PV (horizontal red channel). As a negative control for colocalization (B) shows examples of Syt2 positive innervations with VGLUT2 or CR. Adjacent are scatter plots showing distribution of fluorescence intensities for Syt2 (vertical green channel) and VGLUT2 or CR (horizontal red channel). (C) The average Manders' coefficients of all the scatter plots. Coefficient values in different cortical areas are similar to V1 for colocalization of Syt2 and PV, and differ strongly from non-colocalization values for Syt2 with VGLUT2 or CR (n = 8; n = 7&8). M1, primary motor cortex (n = 6&9); S1, primary somatosensory cortex (n = 7&10); S1BF, barrel field cortex (n = 11&11); Au1, auditory cortex (n = 4&9). Data in C are given in mean coefficients ± SEM. Scale bar 10 µm.

To confirm that the levels of colocalization between Syt2 and PV in other cortical areas were similar to those in V1 we performed scatter plot pixel analyses. Neurons with perisomatic localization of Syt2 puncta in L2-3 and L5 of primary motor cortex, primary somatosensory cortex, barrel field cortex and primary auditory cortex were selected and compared to primary visual cortex. Scatter plot analyses of all areas compared to visual cortex showed no large differences in colocalization between Syt2 and PV assessed by Manders' coefficients (Fig. 6A&C). As expected, Manders' coefficients for colocalization between Syt2 and VGLUT2 or CR in V1 were much lower than those for Syt2 and PV in any cortical area (Fig. 6B&C).

### Syt2 is expressed before PV in mouse visual cortex

**Figure 7:**
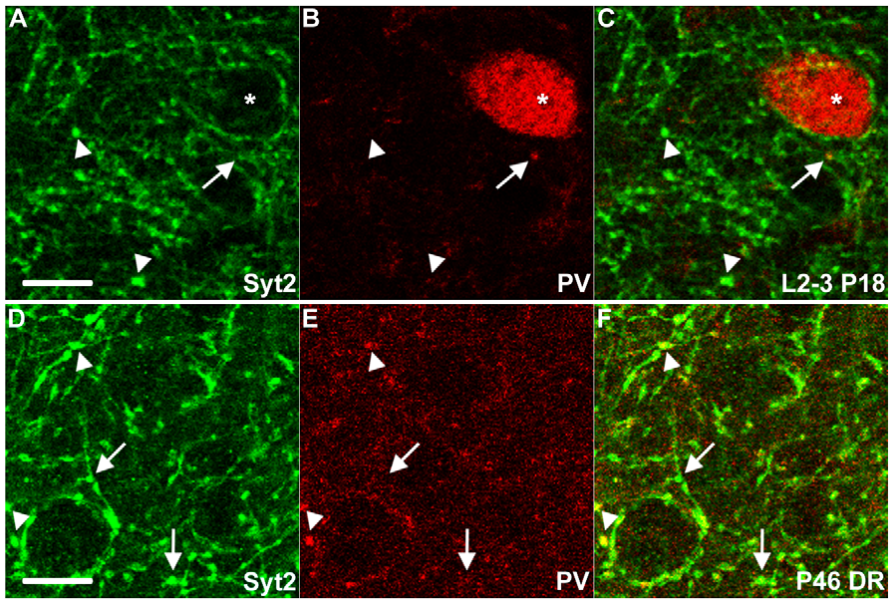
Syt2 and PV expression at P18 and with dark-rearing. (A–C) In L2-3 of the mouse visual cortex Syt2 is expressed in synaptic boutons at P18 when expression of PV is still hardly detectable in neurites or boutons. Arrowheads indicate Syt2 boutons without PV expression. The arrow points to an emerging PV punctum that also expresses Syt2. (D–F) In L2-3 of the visual cortex of dark reared mice from birth to P46 Syt2 expression is not reduced at P46 (D) while PV expression is reduced and not clearly localized in perisomatic boutons (compare to Fig. 2J–L) at the same synapses (E&F). The arrowheads indicate two boutons that both express PV and syt2, but he arrows point to two boutons that are not positive for PV. The asterisk marks a PV+ soma. A–C n = 2; D–F n = 3. Scale bar 10 µm.

**Figure 8:**
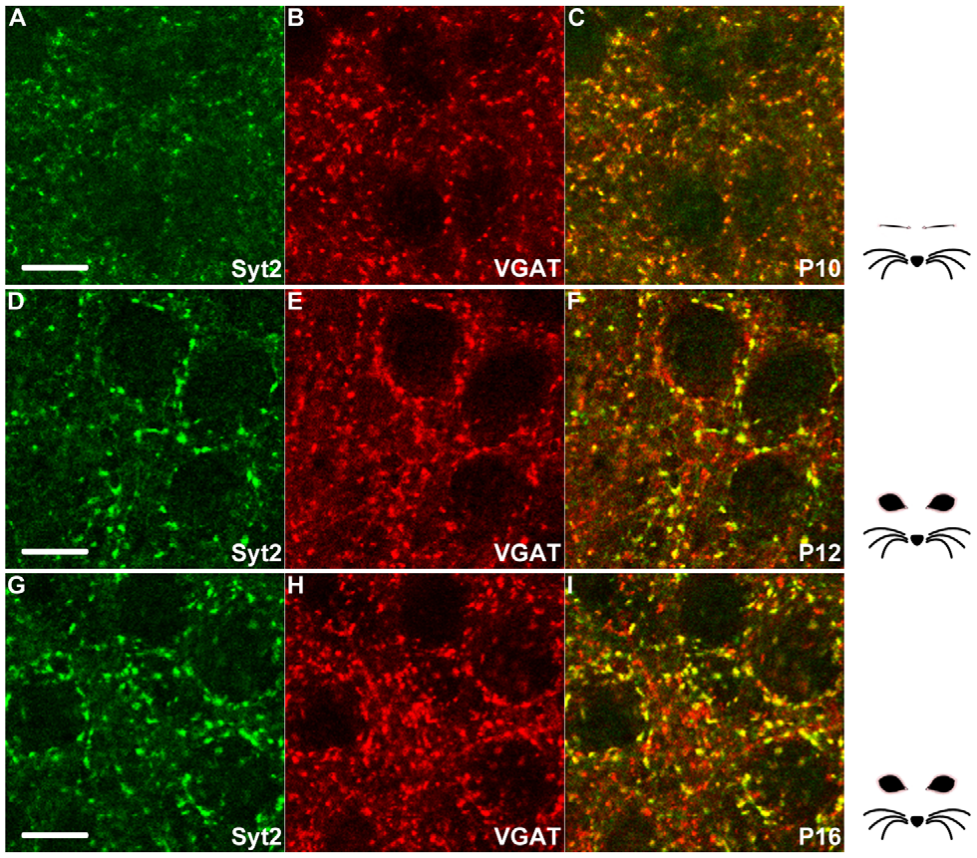
Syt2 is expressed around eye-opening in VGAT positive boutons. (A–C) Before eye-opening Syt2 colocalizes with VGAT+ boutons in L2-3 of the visual cortex. This colocalization continues after eye-opening, but well before onset of PV expression (D–I, and also Fig. 7B). A–C n = 3; D–F n = 2; G–I n = 3. Scale bar 10 µm.

Expression of PV is known to become detectable around two-to-three weeks after birth, which is later than most other interneuron markers. PV expression is initially only detectable in cell bodies but is later also clearly discernable in dendrites and axons [12], [32]. These properties cause difficulties for the analysis of the development of boutons of PV+ neurons, as it is impossible to differentiate increases in PV expression levels from increases in the numbers of synapses formed by PV+ interneurons. Moreover, the high expression levels of PV in the soma make it impossible to visualize PV+ boutons innervating PV+ interneurons. We therefore tested whether Syt2 can be detected before PV expression and whether it can be used to detect innervation of PV interneurons by PV+ boutons. We found that Syt2 was already highly expressed in the visual cortex of P18 mice when PV was still barely detectable (Fig. 7A, B, C). Using similar microscope settings as for the previous experiments, the perisomatic localization of Syt2 onto neurons was clearly detectable in L2-3 while almost no PV immunolabeling could be observed (Fig. 7A, B, C). Since Syt2 is expressed before any strong PV expression is discernable we wanted to check if Syt2 was already expressed around eye-opening. Indeed at P10 a subset of VGAT positive boutons expressed Syt2 when the eyes were still closed (Fig. 8A, B, C). These boutons that were both positive for VGAT and Syt2 continued to be present at P12 and P16 after eye opening (Fig. 8D, E, F, G, H, I). When observing PV+ interneurons, clear Syt2+ puncta could be seen around the soma (Fig. S3). These puncta were PV+ but difficult to discriminate from the PV+ soma. The co-labeling of these puncta by both PV and Syt2 aids the visualization of these innervations. We conclude that Syt2 is an excellent marker for studying the development of synapses formed by PV+ interneurons.

### Syt2 expression is not down-regulated in the visual cortex of dark-reared mice

As PV expression is strongly regulated by neuronal activity, analysis of the effects of dark rearing or monocular deprivation on synapse formation by PV+ interneurons is difficult to quantify. We therefore wanted to determine whether Syt2 is similarly affected by visual experience, or whether it is a more reliable structural marker. We found that in mice dark reared until P46, Syt2 in L2-3 was expressed at comparable levels as juvenile or adult mice (Fig. 7D). In contrast, a strong decrease in PV expression could be observed after dark rearing as seen before in the mouse visual cortex [16] (Fig. 7E). Especially the expression of PV in synaptic boutons was reduced with dark rearing while Syt2 was still clearly present (Fig. 7F). We conclude that Syt2 is an excellent marker for boutons from PV+ interneurons also in the dark reared visual cortex.

## Discussion

The activity-dependent development of synapses formed by PV+ interneurons is believed to be one of the crucial regulators of sensitive period onset in the visual cortex. Unfortunately, PV is not a reliable marker for these synapses as its expression is not restricted to synaptic boutons, and varies with development and visual experience. In this study we identify a more reliable marker for these synapses, Syt2. We found that Syt2 immunolabeling in the visual cortex showed a clear punctate labeling that was often perisomatically organized. It was only found in neuronal structures also expressing PV and VGAT, a vesicular GABA transporter expressed in inhibitory boutons. We did not detect noteworthy colocalization of Syt2 with calbindin, calretinin, somatostatin or with glutamatergic bouton markers VGLUT1 or 2. The colocalization of Syt2 with PV seemed to be constant throughout the cortex. We conclude that Syt2 is a reliable marker for PV+ synaptic boutons in the visual cortex.

In previous studies the expression of Syt2 in PV positive interneurons was also observed in the hippocampus [25], [33]. However, in this brain area the extent of colocalization appears to be less complete. Using single cell PCR it was found that approximately 30–40% of all PV expressing basket cells in the hippocampus were Syt2 positive [33]. In hippocampal cultures it was found that while most Syt2 puncta were PV positive, PV-negative puncta were also observed [25]. In non-cortical brain areas, Syt2 is not restricted to inhibitory synapses. For example, the Calyx of Held in the medial nucleus of the trapezoid body is also positive for Syt2 despite the fact that it is glutamatergic [34], [35]. Interestingly, this synapse does express PV [34], [35]. In the retina, Syt2 is found in horizontal cells, which are GABAergic, but also in off-bipolar cells which are glutamatergic [24].

PV+ interneurons in the forebrain are predominantly fast-spiking cells. Also the Calyx of Held, albeit glutamatergic, is fast-spiking. The preferential use of Syt2 over Syt1 in these fast-spiking neurons may thus have a functional implication. Similarly to Syt1, Syt2 acts as a Ca^2+^ sensor and triggers the evoked release after an action potential and constrains the spontaneous release of neurotransmitter [21]. Syt2 has a slightly lower affinity for Ca^2+^ than Syt1 [36], [37], [38] which may allow for faster dynamics, and thus faster firing [21], [22]. PV is a strong but slow Ca^2+^ buffer and within the Ca^2+^ nanodomain [39], [40] it could therefore cooperate ideally with a fast Ca^2+^ sensor like Syt2 to quickly register increases in Ca^2+^ levels, activating the vesicle release complex to mediate a fast discharge of GABA.

We found that Syt2 in the mouse visual cortex is already expressed at high levels at P18 when PV expression is still very low and difficult to detect in synaptic boutons. Syt2 was even expressed before (P10) and after (P12, P16) eye-opening in VGAT+ boutons. This suggests that the inhibitory innervation by interneurons expressing PV is present before the protein can be detected in their axons and exemplifies the importance of a marker for synapses formed by these cells. Moreover, we found that in the visual cortex of dark reared mice it is difficult to detect PV expression in synaptic boutons, while Syt2 expression levels are unaffected. These properties of Syt2 make it possible to use it as a marker for studying the development and experience dependent changes in inhibitory synapses formed by PV+ interneurons. As Syt2 is an important component of the release-machinery in synaptic terminals of these interneurons, it is well possible that the intensity of the Syt2 staining in individual boutons correlates with their synaptic efficacy, making it an even more useful marker.

PV expressing interneurons form extensive networks with each other, enabling them to detect synchronous neuronal inputs with high sensitivity and to coordinate the firing of large sets of excitatory neurons that they innervate. It is believed that this control of neuronal synchrony is crucial for information processing and synaptic plasticity in the visual cortex [7], [10], [41]. It is therefore important to not only study the experience-dependent development of PV interneuron mediated perisomatic innervation of excitatory neurons but also that of PV neurons themselves. These synapses cannot be visualized by PV antibodies alone, as it is not possible to distinguish the PV+ boutons from the PV+ cell body they innervate. However, if Syt2 and PV immunolabeling are combined, boutons from one PV interneuron terminating onto another PV interneuron will be double labeled and clearly distinguishable. In this study Syt2 puncta could easily be detected around the somata of other PV+ interneurons (Fig. S3).

We conclude that Syt2 is an excellent marker for synaptic boutons of PV+ interneurons in the visual cortex. Its expression is high during development and not down-regulated by dark rearing. Together these properties greatly facilitate the analysis of the experience-dependent development and plasticity of inhibitory inputs from PV+ interneurons onto excitatory and inhibitory neurons, which are important events in the regulation of sensitive period plasticity. Finally, the specific presence of Syt2 in PV+ boutons in the visual cortex may also have functional implications for how their development regulates sensitive period and it would be highly interesting to study whether and how Syt2 deficiency may affect this process.

## Materials and Methods

### Ethics Statement

All experiments involving mice were approved by the institutional animal care and use committee of the Royal Netherlands Academy of Arts and Sciences. On the 20th of May 2010 the academic institutional animal care and use committee (DEC) has declared that the importance of the research balances the use and inconvenience of the animals involved. Furthermore the committee thinks that the conduct of the research does not conflict with any other ethical considerations concerning the use of animals and that there are no realistic alternatives to reach the scientific goals described in this research protocol. The scientific quality has been approved prior to the handling of this research proposal by the DEC in the usual academic fashion. The committee adjudged this proposal as ‘positive’ for the duration of 2 years under the protocolnumber: NIN1036.

### Mice

Male and female C57BL/6J mice (P10, P12, P16, P18, P46, P50–P60) were housed on a standard 12 h light-dark cycle under controlled temperature. For tissue dissection, mice were injected with an overdose (0.3–0.5 ml) Nembutal (Janssen, The Netherlands) and perfused with 0.01 M cold PBS following 80 ml of 4% paraformaldehyde in 0.01 M PBS. Mouse brains were post-fixated for 2 hours. Coronal 50 µm sections were made using a vibratome (VT1000S, Leica, Rijswijk, The Netherlands). To allow long-term storage 10% SodiumAzide (NaN3) was added to the sections, but generally sections were stained soon after sectioning. For some experiments mice were housed in the dark from before birth until perfusion at P46. Sacrificing of these animals was also performed in the dark.

### Immunohistochemistry

Free floating mouse brain sections were incubated with primary antibodies in 0.01 M PBS (pH 7.4) with 5% normal goat serum and 0.1% Triton X-100 overnight at 4°C after 2 hours blocking in medium without antibodies. For Syt2 we made use of a polyclonal rabbit antibody (1∶1000, i735) [17]. Other antibodies were monoclonal mouse anti-PV antibody (1∶1000, Swant, Bellinzona, Switzerland), rat anti-somatostatin (1∶100, Millipore, Temecula, CA, US), mouse anti-calretinin (1∶250, Swant, Bellinzona, Switzerland), mouse anti-calbindin D-28k (1∶4000, Swant, Bellinzona, Switzerland),mouse anti-VGAT (1∶1000, SySy, Göttingen, Germany), mouse anti-VGLUT2 (1∶500, Millipore, Temecula, CA, US) and guinea-pig anti-VGLUT1 (1∶1300, Millipore, Temecula, CA, US) with biotinylated goat anti-guinea-pig (Vector Laboratories, Burlingame, CA, US) and Cy3-labeled Streptavidin (Jackson IR Laboratories, West Grove, PA, US). Except for VGLUT1 all antibodies were visualized by incubating the sections in goat anti-mouse, goat anti-rat, or goat anti-rabbit IgG Alexa Fluor 568 or 488 conjugated secondary antibodies (1∶1000, Molecular Probes, Grand Island, NY, US) for 1 hour at room temperature followed by three rinses in 0.01 M PBS with 0.1% Tween 20 for 10 minutes and once more with PBS without Tween. Sections were then mounted in Mowiol and glass covered for further imaging.

### Fluorescent microscopy

Stained sections were imaged using an Axioplan 2 Zeiss microscope with a HAL lower illumination and an XB075W/2 upper illumination Arc lamp (Carl Zeiss Benelux, Sliedrecht, The Netherlands) for overview images and to localize different brain areas. Immunoreactive puncta were imaged using a Carl Zeiss CLSM 510 Meta confocal microscope (Zeiss) with Argon (488 nm) and HeNe (543 nm) lasers (Carl Zeiss Benelux, Sliedrecht, The Netherlands) and a 40× Carl Zeiss Oil-Objective. For each image acquisition session, the laser intensity and detector gain were adjusted so that the detector range was used without saturation in the puncta.

### Puncta analysis

Puncta were analyzed using a custom made FluorCounter macro for Image-Pro PLUS (v6.3). Puncta were randomly selected with a circular region of interest (ROI) of 3 µm diameter in the red channel and the green channel separately and consecutively, to allow unbiased intensity measurements of both channels per image. Approximately 30–40 puncta per channel were analyzed per image.

First, for the red channel the lower 10th percentile of the ROI intensity values were determined. Second, for each ROI, intensities for the red channel in an adjacent region right next to a punctum were used as background values. Then, intensity thresholds were calculated by averaging the lower 10th percentile of the ROI intensity values with the sum of the average background values plus 2 standard deviations of the average background value. The same procedure was done for the green channel

For both channels, the intensity value in a punctum was compared with the calculated intensity threshold for the same channel of the same image. Any values above intensity threshold were considered as true signal. Percentages of puncta per image with colocalization were used to calculate average colocalization with a standard error of the mean (SEM).

### Pixel analysis

Areas of interest (AOI) of fluorescent images of 100×100 pixels were analyzed in Image-Pro PLUS (v6.3) and generated scatter plots for intensities of both channels. For each channel thresholds were set and Manders' coefficients calculated [42]. Manders' coefficients estimate the contribution of one channel in the areas of the image above thresholds to the overall colocalized fluorescence in the image. M1 is used to describe the contribution of green to the colocalized area while M2 is used to describe the contribution of red.

## Supporting Information

Figure S1 **Syt2 in L4 and L6 is expressed in PV positive boutons.** (A–F) Syt2 almost always colocalizes with PV in L4 and L6 of the visual cortex. (G) Percentages colocalization of PV in Syt2+ puncta (PV/Syt2) and Syt2 in PV+ puncta (Syt2/PV). A–F n = 4. Scale bars 10 µm. Data in G are given in mean percentage ± SEM.
(TIF)

Figure S2 **Syt2 is not expressed in PV positive neuritis.** (A–C) The presynaptic protein Syt2 is only expressed in PV puncta and not in PV positive neurite stretches delineated with the white boxes in the examples in C. In (B) neurites are visible as stretches of PV positive structures, that are devoid of Syt2 labeling (A). Magnifications of the neurite in the closed box are shown below. Scale bar 20 µm.
(TIF)

Figure S3 **Syt2 is expressed in PV positive perisomatic boutons on PV positive neurons.** (A–C) Example of a PV expressing neuron with perisomatically localized boutons expressing both PV and Syt2. PV positive boutons are almost indistinguishable from the somata (arrowheads, B). Syt2 boutons are perisomatically arranged in the same boutons containing PV (A&C) as indicated by the white arrowheads. Scale bars 10 µm.
(TIF)
