# Correction: Synaptotagmin-2 Is a Reliable Marker for Parvalbumin Positive Inhibitory Boutons in the Mouse Visual Cortex

**DOI:** 10.1371/annotation/1c5484e5-41c0-44dc-8422-2dbd3a002f3b

**Published:** 2012-07-10

**Authors:** Jean-Pierre Sommeijer, Christiaan N. Levelt

The legends for Figures 4 and 5 were incorrectly switched. The legend that appears with Figure 4 should be with Figure 5, and the legend that appears with Figure 5 should be with Figure 4. The figure images appear in the correct order. 

