# Supplementary figures and images for: Synaptotagmin-2 Is a Reliable Marker for Parvalbumin Positive Inhibitory Boutons in the Mouse Visual Cortex
Source: PLoS One. 2012 Apr 23;7(4):e35323. doi: 10.1371/journal.pone.0035323 (PMC3335159; doi:10.1371/journal.pone.0035323)

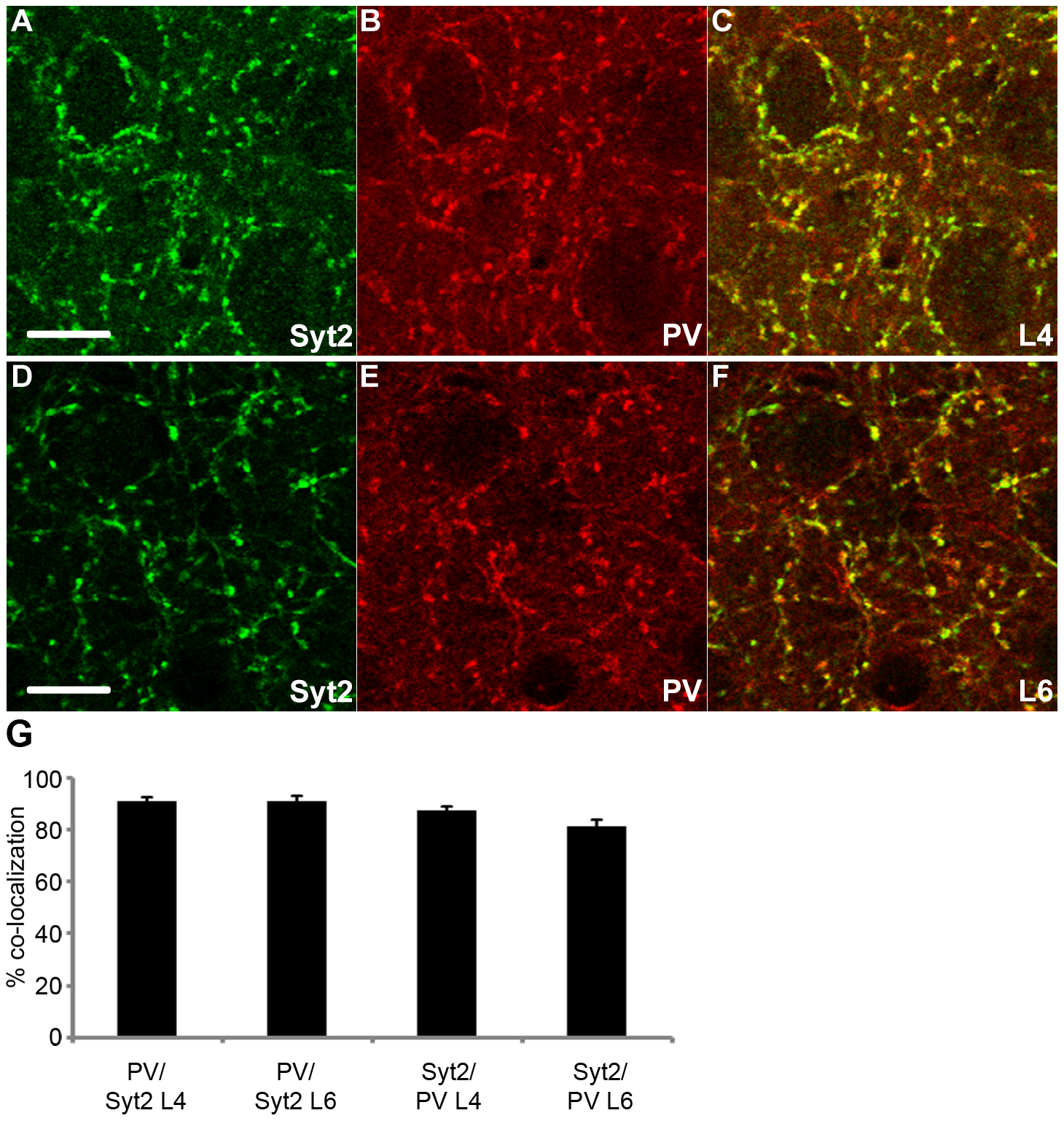

Supplement: Figure S1 — Syt2 in L4 and L6 is expressed in PV positive boutons. (A–F) Syt2 almost always colocalizes with PV in L4 and L6 of the visual cortex. (G) Percentages colocalization of PV in Syt2+ puncta (PV/Syt2) and Syt2 in PV+ puncta (Syt2/PV). A–F n = 4. Scale bars 10 µm. Data in G are given in mean percentage ± SEM. (TIF) [file pone.0035323.s001.tif]

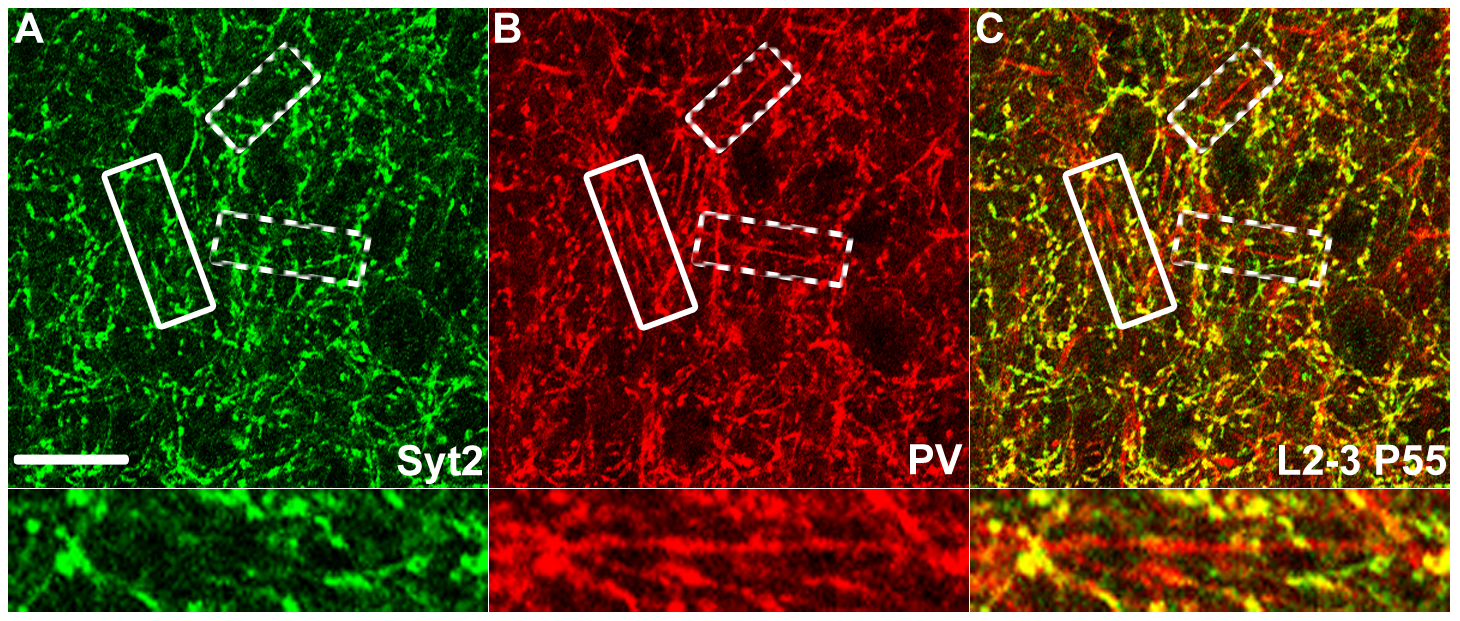

Supplement: Figure S2 — Syt2 is not expressed in PV positive neuritis. (A–C) The presynaptic protein Syt2 is only expressed in PV puncta and not in PV positive neurite stretches delineated with the white boxes in the examples in C. In (B) neurites are visible as stretches of PV positive structures, that are devoid of Syt2 labeling (A). Magnifications of the neurite in the closed box are shown below. Scale bar 20 µm. (TIF) [file pone.0035323.s002.tif]

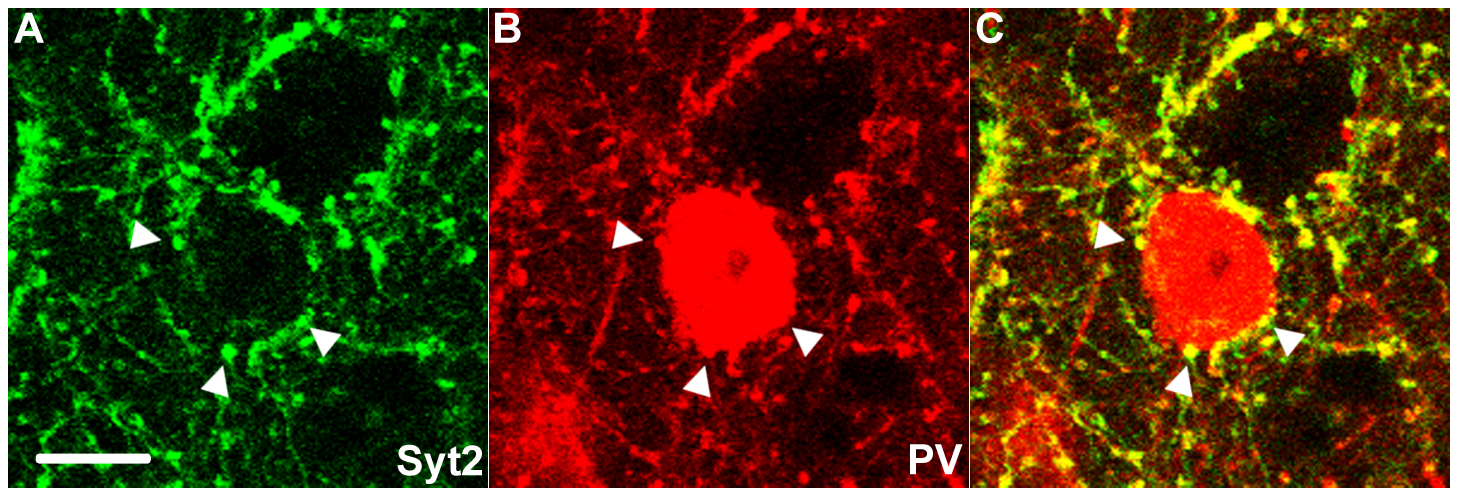

Supplement: Figure S3 — Syt2 is expressed in PV positive perisomatic boutons on PV positive neurons. (A–C) Example of a PV expressing neuron with perisomatically localized boutons expressing both PV and Syt2. PV positive boutons are almost indistinguishable from the somata (arrowheads, B). Syt2 boutons are perisomatically arranged in the same boutons containing PV (A&C) as indicated by the white arrowheads. Scale bars 10 µm. (TIF) [file pone.0035323.s003.tif]
